# Alterations of mesenchymal stromal cells in cerebrospinal fluid: insights from transcriptomics and an ALS clinical trial

**DOI:** 10.1186/s13287-021-02241-9

**Published:** 2021-03-18

**Authors:** Ashley A. Krull, Deborah O. Setter, Tania F. Gendron, Sybil C. L. Hrstka, Michael J. Polzin, Joseph Hart, Amel Dudakovic, Nicolas N. Madigan, Allan B. Dietz, Anthony J. Windebank, Andre J. van Wijnen, Nathan P. Staff

**Affiliations:** 1 Department of Neurology, Mayo Clinic, 200 First St. SW, Rochester, MN 55905 USA; 2 Department of Neuroscience, Mayo Clinic, Jacksonville, FL 32224 USA; 3 Department of Orthopedic Surgery, Mayo Clinic, Rochester, MN 55905 USA; 4 Department of Laboratory Medicine and Pathology, Mayo Clinic, Rochester, MN 55905 USA

**Keywords:** Mesenchymal stromal cell, Gene expression, Neuroinflammation, Growth factors, Cerebrospinal fluid, Amyotrophic lateral sclerosis, Human studies

## Abstract

**Background:**

Mesenchymal stromal cells (MSCs) have been studied with increasing intensity as clinicians and researchers strive to understand the ability of MSCs to modulate disease progression and promote tissue regeneration. As MSCs are used for diverse applications, it is important to appreciate how specific physiological environments may stimulate changes that alter the phenotype of the cells. One need for neuroregenerative applications is to characterize the spectrum of MSC responses to the cerebrospinal fluid (CSF) environment after their injection into the intrathecal space. Mechanistic understanding of cellular biology in response to the CSF environment may predict the ability of MSCs to promote injury repair or provide neuroprotection in neurodegenerative diseases.

**Methods:**

In this study, we characterized changes in morphology, metabolism, and gene expression occurring in human adipose-derived MSCs cultured in human (hCSF) or artificial CSF (aCSF) as well as examined relevant protein levels in the CSF of subjects treated with MSCs for amyotrophic lateral sclerosis (ALS).

**Results:**

Our results demonstrated that, under intrathecal-like conditions, MSCs retained their morphology, though they became quiescent. Large-scale transcriptomic analysis of MSCs revealed a distinct gene expression profile for cells cultured in aCSF. The aCSF culture environment induced expression of genes related to angiogenesis and immunomodulation. In addition, MSCs in aCSF expressed genes encoding nutritional growth factors to expression levels at or above those of control cells. Furthermore, we observed a dose-dependent increase in growth factors and immunomodulatory cytokines in CSF from subjects with ALS treated intrathecally with autologous MSCs.

**Conclusions:**

Overall, our results suggest that MSCs injected into the intrathecal space in ongoing clinical trials remain viable and may provide a therapeutic benefit to patients.

**Supplementary Information:**

The online version contains supplementary material available at 10.1186/s13287-021-02241-9.

## Introduction

Mesenchymal stromal cells (MSCs) are increasingly being employed in clinical trials to treat neurodegenerative diseases despite a lack of mechanistic investigations into the phenotypes of MSCs when injected into the central nervous system (CNS) compartment. For many current trials for neurological diseases, MSCs are extracted from adipose tissues or bone marrow, expanded in vitro, and then injected into the intrathecal cerebrospinal fluid (CSF) of the CNS to bypass the blood-brain barrier. The hypothesis, based on pre-clinical in vitro and animal models, is that MSCs will secrete factors that have a therapeutic effect on neurons and the surrounding microenvironment. MSC-based therapy is attractive because most neurodegenerative diseases involve multi-faceted pathologies, and MSCs have been shown to favorably modulate the majority of these pathological processes. However, pre-clinical models often recapitulate neither the intrathecal microenvironment nor the preparation of MSCs that would be administered to a clinical trial patient, hampering responsible translation of MSCs into clinical use.

Intrathecal CSF represents a uniquely challenging environment for cultured MSCs: it has a lower partial pressure of oxygen than media in culture conditions (~ 70–80 mmHg in CSF) [1], undergoes age- and disease-related changes in protein composition [2–4], and contains less protein compared to plasma, with CSF containing primarily albumin and brain-derived factors [5]. When MSCs are cultured in vitro, cells are grown at normoxia and the growth media must be supplemented with nutrient-rich human platelet lysate (hPL) to support healthy proliferation prior to their harvest and implantation. When cultured without serum or hPL, or in hypoxic conditions, MSCs have been shown to rapidly undergo apoptosis and risk chromosomal instability [6–8]. However, previous studies—albeit utilizing diluted CSF formulations—have shown that MSCs can remain viable and increase the presence of nutritional factors within CSF [9–11]. So how are researchers and clinicians to harmonize this data to balance the risks with the purported benefits of MSC therapy?

In addition to the above-mentioned weaknesses of pre-clinical models, they often also fail to account for the preparation of MSCs for clinical use, which has the potential to drastically impact the phenotype of MSCs. The field of cell therapy is becoming increasingly cognizant of how culture conditions affect MSCs. Understandably, most preclinical research to date has been conducted using research-grade reagents, such as media supplemented with fetal bovine serum, that are not permitted for cells destined for human use. This distinction is important to note as numerous studies demonstrated that MSCs grown in compliance with current good manufacturing practices (GMP) versus non-GMP conditions (and even within varying GMP-compliant conditions) display disparate phenotypes characterized by different proliferation rates, differentiation potentials, and immunomodulatory potentials [12–15]. At present, it is unclear how in vitro manufacturing conditions ultimately influence the response of MSCs to the CSF environment. Furthermore, how the CSF microenvironment, with its lower oxygen and nutrient levels, impacts MSCs cultured for clinical use is also unknown.

To address these gaps in understanding, and to guide stakeholders in MSC-based treatment of neurodegenerative diseases, we aimed to characterize the morphological, metabolic, and transcriptomic changes observed when MSCs are incubated in undiluted human (hCSF) or artificial CSF (aCSF) as compared to control media (CM; containing GMP-grade hPL) and platelet lysate-free (PLF) media. We hypothesized that the phenotype of MSCs cultured in undiluted CSF formulations would mimic that of MSCs cultured in conditions devoid of platelet lysate, which are known to be stressful for MSCs. To extend prior work and to most accurately reflect the clinical application, our studies emphasized the use of clinical-grade MSCs cultured in GMP-grade reagents to recapitulate the phenotype of cells that are currently used to treat patients [16–18].

To supplement our in vitro results, we undertook a secondary set of experiments in which we analyzed CSF collected from subjects in a phase I clinical trial at the Mayo Clinic investigating the use of intrathecal MSCs to treat amyotrophic lateral sclerosis (ALS) [17], a neurodegenerative disorder that leads to a progressive and fatal paralysis. These secondary experiments were designed to examine the protein levels of therapeutically-relevant growth and immunomodulatory factors within the CSF of patients treated with MSCs for additional evidence to support the potential therapeutic mechanism of action of MSCs within the intrathecal space.

## Materials and methods

### Mesenchymal stromal cells and cerebrospinal fluid

With approval from the Mayo Clinic Institutional Review Board (IRB), MSCs were obtained from four consenting, healthy patients who underwent elective removal of subcutaneous adipose tissue either by lipoaspiration [male/41 years (M1), female/32 years (F1), male/54 years (M2)] or by surgical biopsy [a female (F2) bariatric patient who donated cells as medical waste so age was not collected]. Samples were processed to isolate and expand MSCs as previously described [7, 19, 20]. Cells were stored in liquid nitrogen until use. All patient information was kept confidential and all identifiers removed prior to any studies.

hCSF that otherwise would be disposed as medical waste was obtained from consenting patients treated with lumbar puncture in the Hydrocephalus Clinic at the Mayo Clinic with approval from the Mayo Clinic IRB.

### Solutions

CM used for studies consisted of Advanced Minimal Essential Medium (MEM) standard culture medium with 5% PLTmax (platelet lysate solution, Mill Creek Life Sciences), 2 U/mL heparin (hospital pharmacy), 2 mM L-glutamine (Invitrogen), and antibiotics (100 U/ml penicillin, 100 g/ml streptomycin). The PLF media was made using Advanced MEM with 2 mM L-glutamine and antibiotics. The aCSF formulation comprised the following: distilled water with NaCl (124 mM), KCl (2.5 mM), NaHCO_3_ (26 mM), NaH_2_PO_4_-H_2_O (1.25 mM), MgSO_4_ (1 mM), CaCl_2_ (2 mM), D-glucose (60 mg/dL), and human albumin (30 mg/dL). The aCSF had a pH of 7.4 and was warmed along with the other solutions before use.

### Immunocytochemistry on MSCs cultured in human CSF

MSCs were seeded in CM at 3000 cells/cm^2^ in 6-well plates for 24 h on glass coverslips prior to introduction of fresh CM, PLF media, or hCSF for 24–48 h. Upon removal of the media, cells were stained with pre-warmed 100 nM MitoTracker CMX-Ros solution (Invitrogen, Carlsbad, CA) for 20 min at 37 °C. Cells were then fixed for 10 min in a pre-warmed solution of 4% paraformaldehyde in Dulbecco’s phosphate buffer solution (D-PBS). Following fixation, cells were washed 3x for 5 min in 1x D-PBS, permeabilized for 10 min with phosphate buffered solution with Triton X-100 (PBS-T—0.1% Triton X-100 in 1x PBS), and washed again 3x for 5 min in D-PBS. Cells were blocked for 30 min with 1% bovine serum albumin in 1x D-PBS. Cells were incubated for 60 min with Alexa Fluor 488 phalloidin (1:100, Invitrogen, Carlsbad, CA). After washing in 1x D-PBS and removal of excess liquid, the cover slips were mounted onto slides using ProLong Gold antifade reagent with DAPI (Invitrogen, Carlsbad, CA) and left to cure overnight. Slides were imaged with an AxioScope fluorescent microscope at × 40 magnification.

### Metabolic activity of MSCs in aCSF

MSCs were seeded in CM at 2500 cells/cm^2^ in 12-well plates and grown to about 80% confluency prior to introduction of fresh CM, PLF media, or aCSF for 24–96 h. At each time point, culture solutions were collected and cell viability analyzed using an MTS assay following the manufacturer’s instructions (Promega, Madison, WI). Absorbance of each sample was read at 490 nm using a plate reader and corrected using absorbance values of empty control wells. Metabolic activity for PLF media- and aCSF-treated cells was assessed in comparison to the absorbance of wells containing control CM-treated cells.

### Transcriptomic analyses

MSCs were seeded with about 3000 cells/cm^2^ in 6-well plates and monitored until approximately 80% confluent. Cells were incubated in with fresh CM, PLF media, or aCSF for 24 or 48 h before harvesting. Cells from each line were plated in three replicate wells per condition per time point to yield 18 wells total per line—6 treated with CM, 6 treated with PLF media, 6 treated with aCSF—with half the wells treated for 24 h and half treated for 48 h. Total RNA was isolated per the manufacturer’s instructions using the miRNeasy Mini Plus Kit (Qiagen, Germantown, MD). RNA libraries were prepared according to the manufacturer’s instructions for the TruSeq RNA Sample Prep Kit v2 (Illumina, San Diego, CA) that uses oligo dT magnetic beads to enrich poly-A mRNA. Polymerase chain reaction (PCR) was used to enrich the resulting cDNA fragments using Illumina TruSeq PCR primers. The concentration and size distribution of the completed library was determined using a Fragment Analyzer (AATI, Ankeny, IA) and Qubit fluorometry (Invitrogen, Carlsbad, CA). The cDNA library was sequenced at 30–50 million fragment reads per sample following Illumina’s standard protocol using the Illumina cBot and HiSeq 3000/4000 PE Cluster Kit. The flow cells were sequenced as 100 X 2 paired-end reads on an Illumina HiSeq 4000 using the HiSeq 3000/4000 sequencing kit and HD 3.4.0.38 collection software. Base-calling was performed using Illumina’s RTA version 2.7.7. In order to validate the transcriptomic PCR data, an independent set of experiments were performed using conventional real-time qPCR and were confirmatory (Supplemental Figure 3).

### Bioinformatics analysis

Gene expression counts were obtained using the MAP-RSeq v.1.2.1.5 workflow [21], the Mayo Bioinformatics Core pipeline. MAP-RSeq consists of alignment with TopHat 2.0.6 [22] against the human hg19 genome build and gene counts with the HTSeq software (http://www-huber.embl.de/users/anders/HTSeq/doc/overview.html). Gene annotation files were obtained from Illumina (https://support.illumina.com/sequencing/sequencing_software/igenome.html). Normalized gene counts were obtained from MAP-RSeq, where expression levels for each gene were normalized to one million reads and corrected for gene length (fragments per kilobase pair per million mapped reads [FPKM]). For heatmap and principal component analysis (PCA) visualizations, significant genes were selected based on an average FPKM > 1, a log-fold-change >or < absolute value of 1, and a *p* value < 0.05, when comparing aCSF-treated to PLF media-treated MSCs. Gene expression values (FPKM) were log transformed and *z*-scaled by gene prior to plotting. Visualizations were constructed in R using the ComplexHeatmap [23] and factoextra (https://github.com/kassambara/factoextra) packages. A heatmap of genes expressed at FPKM > 1 on average and significantly up- or down-regulated (fold change > 2 or < 0.5, *p* < 0.05) between aCSF-treated and PLF media-treated MSCs was generated using the Broad Institute’s Morpheus tool (https://software.broadinstitute.org/morpheus). Functional annotation analysis was performed using DAVID Bioinformatics Resources 6.8 [24, 25]. Pathway enrichment analysis of MSCs cultured in aCSF and CM was completed using GSEA software (version 4.1.0) [26, 27]. Human_MSigdb_January_13_2021_symbol.gmt and Human_GOALL_no_GO_iea_January_13_2021_symbol.gmt from [http://baderlab.org/Genesets] enabled identification of canonical pathways (hallmark gene sets, BIOCARTA, PID Pathways) and GO gene sets that were enriched in aCSF-treated MSCs [28]. Gene sets with FDR < 0.25 (canonical pathways) or FDR < 0.1 (GO) were considered significant.

### VEGF ELISA

To measure the concentration of VEGF protein secreted in vitro from patient adMSCs, cells were again seeded at a density of 3000 cells/cm^2^ in triplicate, and the media was changed upon reaching ~ 80% confluence to fresh CM, PLF, or aCSF media. The supernatant media was collected and the underlying monolayer was harvested at 24 or 48 h time points. Media and cell pellets were flash frozen on dry ice and stored at -80C for DNA or RNA isolation. VEGF within supernatant media was measured using the Quantikine ELISA kit (R&D Systems, Minneapolis MN USA) according to the manufacturer’s instructions using a two-parameter logistical standard curve. VEGF levels were normalized to total DNA following extraction using DNEasy Blood & Tissue Kit (Qiagen, Valencia, CA, USA) and nanodrop quantification.

### Measurement of analyte concentrations in human CSF

ALS patients enrolled in a previously completed clinical trial (ClinicalTrials.gov #NCT01609283) received a dose of autologous adipose-derived MSCs, and CSF was obtained before treatment and 1 week after treatment for analysis [17]. Analytes in CSF were measured in duplicate using commercially available immunoassays from Meso Scale Discovery according to their protocols. Samples were diluted with assay-specific diluents using recommended dilutions for CSF. If no recommendation was provided, the appropriate dilution was determined through linearity of dilution tests. MMP-1 and GDF-15 concentrations were measured with R-PLEX assays using CSF diluted 1 in 10 or 1 in 50, respectively. MCP-1 concentrations were measured with singleplex V-PLEX assays using CSF diluted 1 in 16. VEGF, PIGF, and bFGF concentrations were measured using a multiplex V-PLEX assay using CSF diluted 1 in 2. Each assay plate contained interplate control samples; interplate coefficient of variations for each assay type ranged from 1.79 to 8.84%. Response values corresponding to the intensity of emitted light upon electrochemical stimulation of the assay plate using the Meso Scale Discovery QUICKPLEX SQ120 were acquired, and analyte concentrations were interpolated using MSD Discovery Workbench software.

### Statistical analysis

GraphPad Prism software was used for statistical analysis. Where appropriate, non-parametric data were analyzed by the Kruskal-Wallis test with Dunn’s test to correct for multiple comparisons.

## Results

### MSCs cultured in 100% human CSF for 24 or 48 h remain morphologically unchanged

**Fig. 1:**
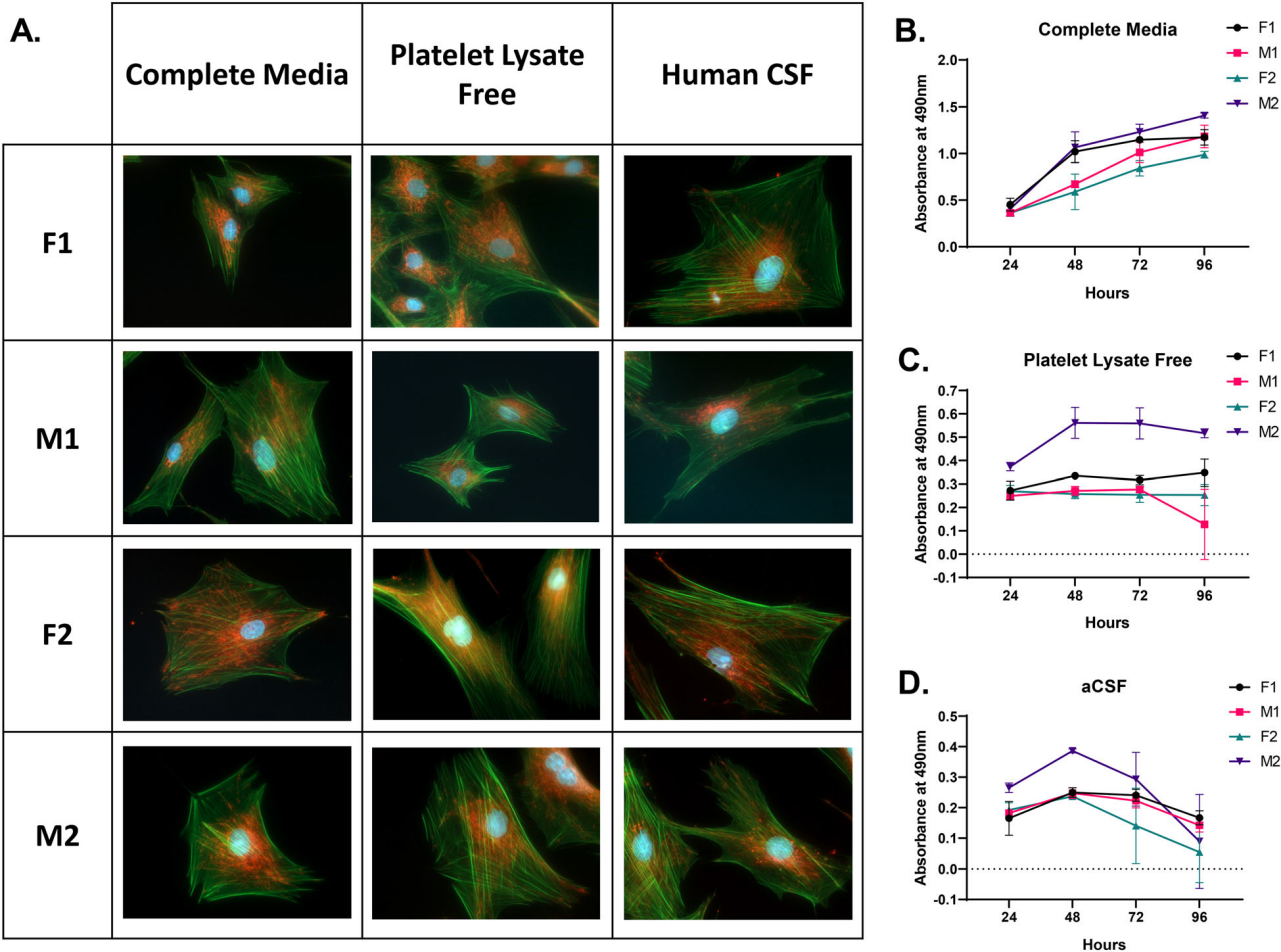
Morphology and viability of MSCs cultured in CM, PLF media, and human or artificial CSF. **a** Morphology of MSCs imaged after 24 h in CM, PLF media, or hCSF. Cells were stained with phalloidin AF488 (green), DAPI (blue), and MitoTracker CMX-Ros (red). Images were taken at 40x magnification. **b** Metabolic activity of MSCs via MTS assay after 24–96 h in CM. **c** Metabolic activity of MSCs via MTS assay after 24–96 h in PLF media. **d** Metabolic activity of MSCs via MTS assay after 24–96 h in aCSF (*n* = 3, mean ± standard deviation)

To analyze the morphological changes of adipose-derived MSCs in CSF, all four lines of MSCs were cultured in CM, PLF media, or hCSF for 24 or 48 h. At each time point, cells were stained with DAPI, phalloidin, and MitoTracker Red CMX Ros to image the nucleus, cytoskeleton, and mitochondria distribution. All of the surviving cells in each condition showed a fibroid-like morphology, with cells spread out in flat monolayers (Fig. 1a). Cells displayed branched cytoplasms and intact cytoskeletons. Mitochondrial distribution was normal, with most mitochondria being concentrated around the nuclei and filopodia. Nuclei appeared elliptical and speckled as expected. Overall, we detected no alterations in cellular morphology between cells cultured in each of the three conditions.

### MSCs exhibit decreased metabolic activity in PLF media and aCSF

We cultured the four lines of MSCs in CM, PLF media, or aCSF for 24–96 h to assess cellular proliferation and compare metabolic activity over time. As expected, cells in CM showed a normal increase in cellular activity, denoting a normal proliferation rate (Fig. 1b). In contrast, cells in nutrient-deprived PLF and aCSF showed a progressive decrease in metabolic activity, most likely due to decreased proliferation and viability (Fig. 1c, d). Rates of metabolic activity loss varied between cell lines, and it is unclear whether the cells remaining after 96 h in aCSF had undergone replication during that time.

### Consistent transcriptomic patterns are dependent on culture condition

**Fig. 2:**
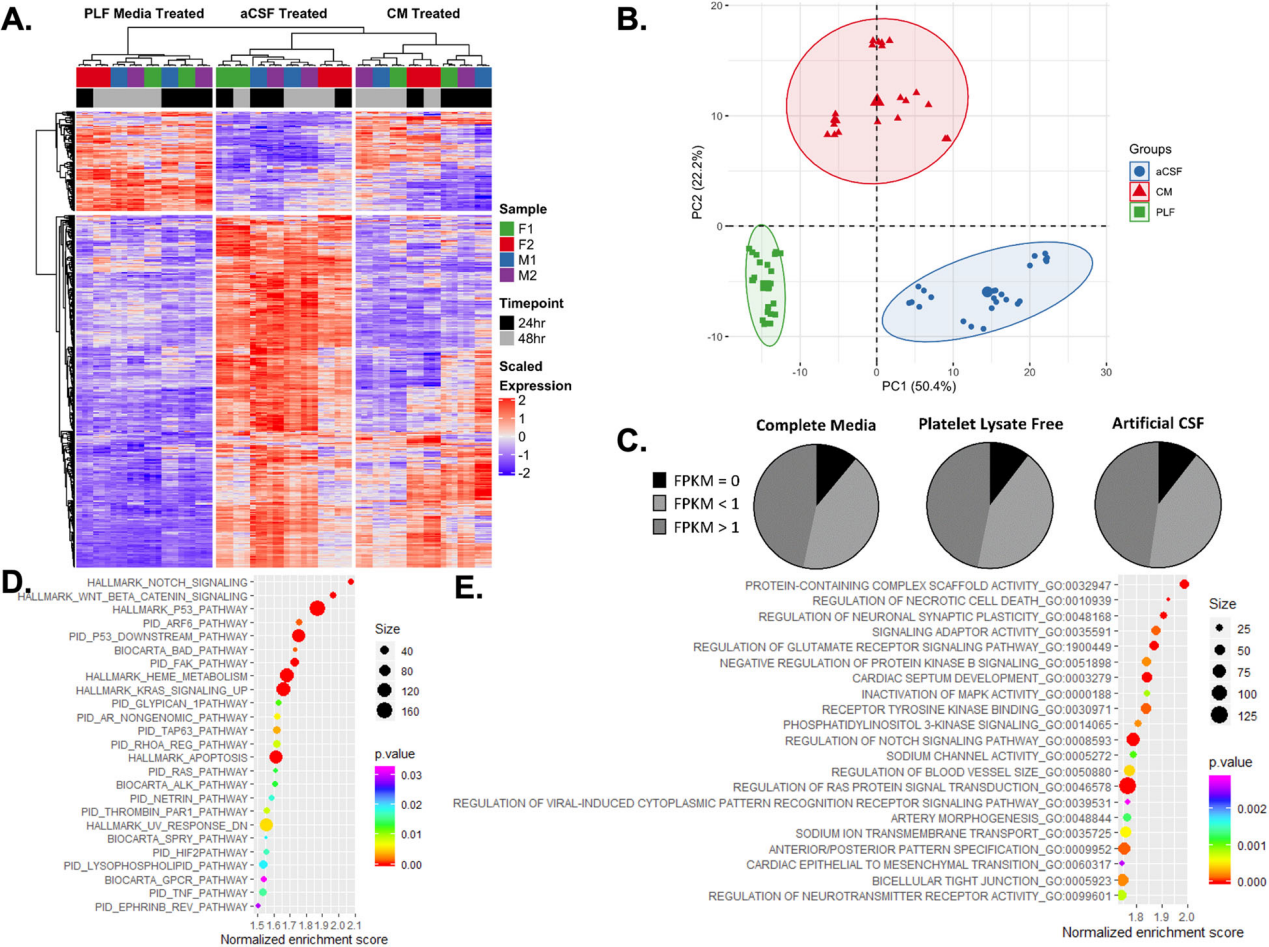
Unbiased transcriptomic analysis reveals homogeneity of biological and technical replicates of MSCs treated with CM, PLF media, or aCSF for 24–48 h. **a** Heatmap of results from samples submitted to RNA sequencing with well-defined clusters of replicates, with the first and second order of separation being dictated by the treatment group and time in the condition. **b** Principal component analysis demonstrates that culture condition and time account for over 50% of the variability in MSC transcriptomes. **c** Breakdown of expression levels of annotated genes for each culture condition. **d** Significantly enriched canonical pathways based on upregulated genes in aCSF-treated MSCs compared to CM-treated MSCs (FDR < 0.25). **e** Topmost enriched GO gene sets based on upregulated genes in aCSF-treated MSCs compared to CM-treated MSCs (FDR < 0.1)

To determine how the transcriptomes of MSCs change in response to CSF, we examined gene expression profiles in the four lines of MSCs cultured in CM, PLF media, and aCSF for 24 or 48 h using high-throughput RNASeq analysis. Unbiased transcriptomic analysis revealed homogeneity of both biological and technical replicates of MSCs cultured in CM, PLF media, or aCSF. Hierarchical clustering produced well-defined clusters of replicates, with the first order of separation being dictated by the treatment group and then by time in each condition (Fig. 2a). Principal components analysis identified culture condition and the time in each condition as accounting for over 50% of the variability between MSCs cultured in each condition (Fig. 2b). The number of annotated genes mapped from RNA reads totaled 23,398 for all conditions. Of these genes, in all conditions almost half of the genes were expressed at levels greater than 1 FPKM, about 10% were not appreciably expressed, and almost 50% were expressed at levels less than 1 FPKM (Fig. 2c).

### MSCs cultured in aCSF maintain gene expression of MSC cell surface markers

**Fig. 3:**
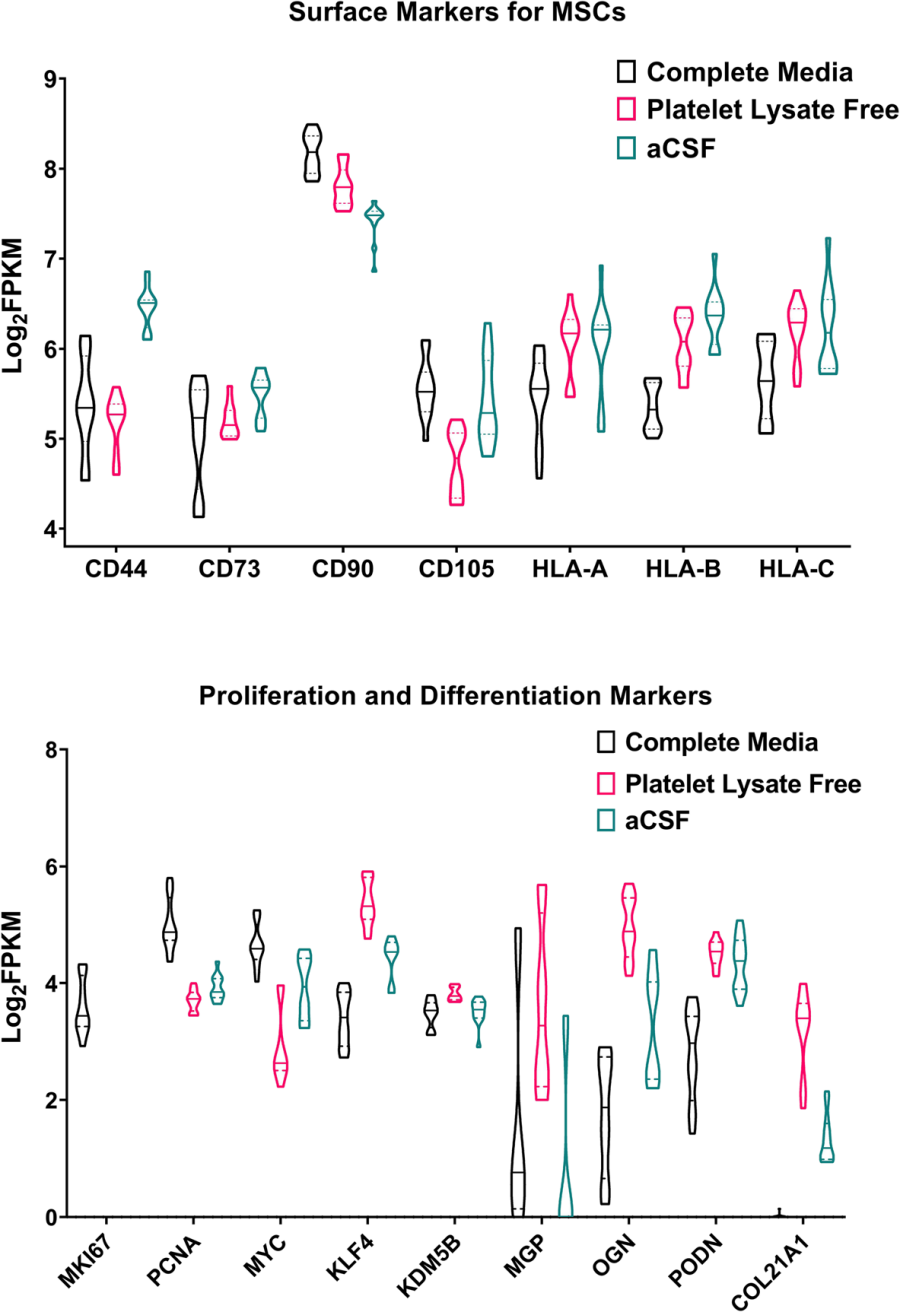
Expression levels of detectable transcripts of genes encoding characteristic MSC markers by MSCs cultured in CM, PLF, and aCSF. **a** Detectable expression levels of transcripts encoding surface markers for MSCs (*n* = 12, data shown with median and interquartile range). **b** Expression levels of transcripts encoding markers of proliferation and differentiation of MSCs. Transcript expression levels are shown as Log_2_FPKM (*n* = 12, data shown with median and interquartile range)

MSCs cultured in aCSF express mRNAs for cell surface markers that have been well-defined for MSCs [20, 29–31]. Standard cell surface markers for MSCs include CD44, CD73/NT5E, CD90/THY1, CD105/ENG, and MHC class 1 molecules. MSCs cultured in aCSF exhibit robust expression of the genes encoding these markers, albeit at varying levels as compared to cells cultured in CM or PLF media (Fig. 3a). Conversely, MSCs cultured in aCSF demonstrate an absence of expression of genes for hematopoietic, stem cell, or endothelial markers (*CD11B*, *CD14*, *CD31*, *CD45*, *CD253A*, *HLA-DRA*) except for *CD34*, which was expressed at very low levels (data not shown).

### MSCs cultured in aCSF decrease expression of genes involved in proliferation and increase expression of genes involved in promoting the non-canonical Wnt signaling pathway

As recommended by the IFATS/ISCT panel [29], we examined expression of genes encoding factors involved in viability and fibroblastoid colony-forming units (CFU-Fs). As compared to cells in CM, cells cultured in PLF media and aCSF do not exhibit expression of *MKI67*, which encodes the proliferation marker Ki67, but retain expression of *PCNA* (Fig. 3b). We also found variable expression of extracellular matrix-associated genes that are reported to be up-regulated in post-proliferative cells (*COL21A1*, *MGP*, *OGN*, *PODN*), suggesting that cells incubated in aCSF are in a unique post-proliferative state [20] (Fig. 3b).

MSCs cultured in all three conditions expressed mRNAs for genes encoding reprogramming factors (*MYC*, *KLF4*, and *KDM5B*) to varying degrees (Fig. 3b). None of the cells cultured expressed genes for markers of pluripotency (e.g., *NANOG*, *POU5F1*, *SOX2*, *LIN28A/B*), corroborating previous observations [20, 32]. Likewise, assessment of expression of lineage-specific genes revealed low-level expression of few genes for adipogenesis (*PPARG*), chondrogenesis (*SOX9*), and osteogenesis (*ALPL*, *RUNX2*), with these genes hypothesized to be playing an alternative role in driving matrix mineralization and cell survival in a post-proliferative state (Supp. Figure 1).

Human primary cell quiescence, in addition to being signaled by cell cycle exit, is also promoted by suppression of the canonical Wnt signaling pathway [33]. Notably, cells incubated in aCSF showed marked increases in genes associated with suppressing the canonical Wnt pathway (*LRP4*, *RAPGEF1*, *AXIN1*, *DKK1*, *DKK3*, *TLE1-4*) and a decrease in *WNT2* expression (Supp. Fig. 2). Interestingly, aCSF-treated cells retained expression of genes encoding Wnt5A, Wnt5B, Wnt7B, and Wnt9A, which activate the non-canonical Wnt pathway to promote osteoblast differentiation and influence synaptic plasticity, which could impact the regenerative effects of MSCs in the CNS [34, 35].

### Expression of genes for secreted factors suggests the response by MSCs to aCSF is immunomodulatory and enriched for growth factors

It is important to assess changes in the expression of genes encoding secreted factors by MSCs in response to aCSF. Secreted factors potentially could serve as biomarkers of MSC activity in vivo, and the expression of genes for secreted growth factors specifically have mechanistic relevance to clinical trials that likely depend on the trophic functions of secreted factors.

**Fig. 4:**
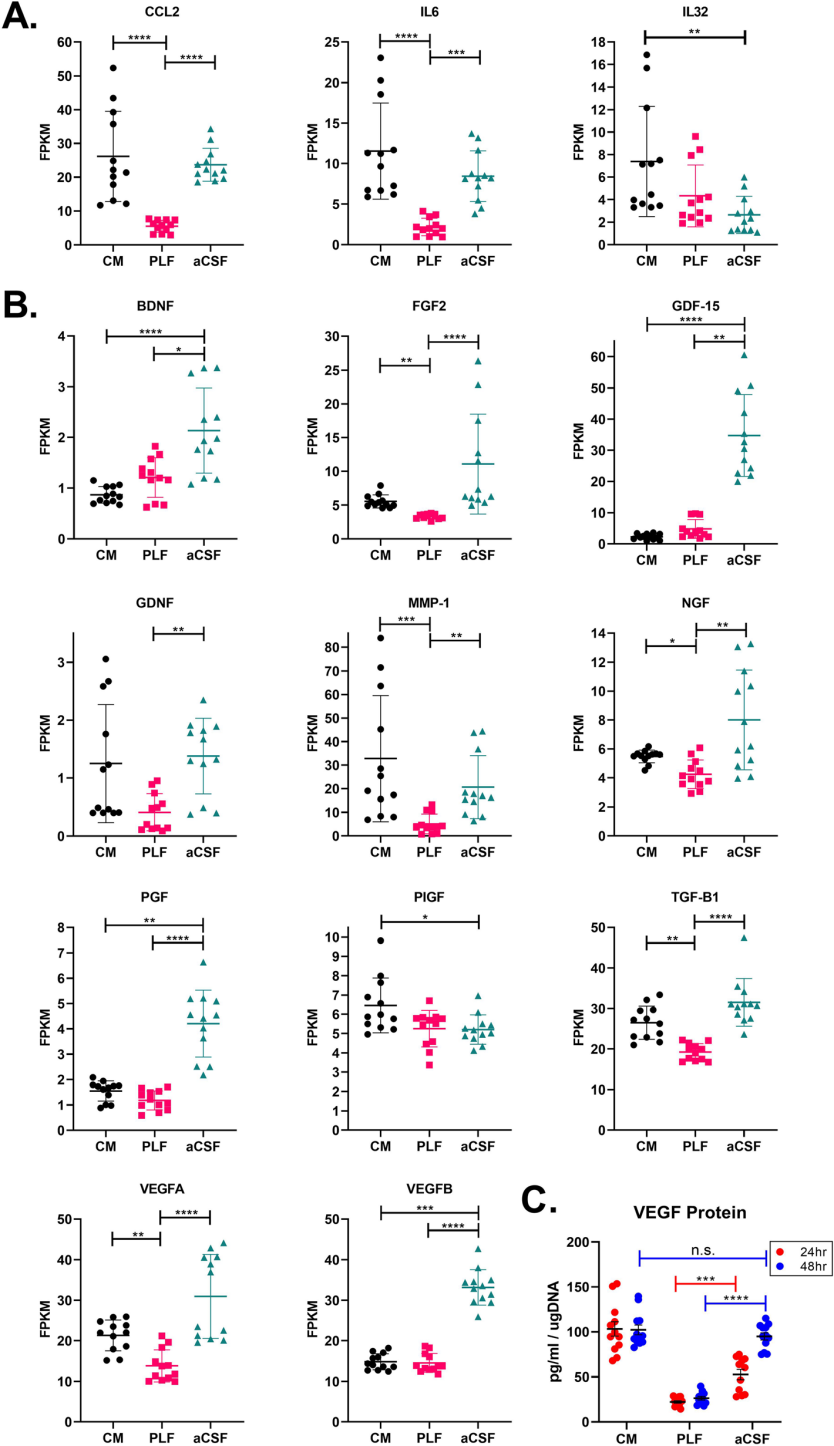
Expression levels of detectable transcripts of genes encoding key immunomodulatory, angiogenic, and neurotrophic factors by MSCs cultured in CM, PLF, and aCSF. **a** Detectable expression levels (FPKM) of genes encoding immunosuppressive and inflammatory factors (*n* = 12). **b** Expression levels (FPKM) of genes encoding select angioneurins and growth factors that are highly expressed by aCSF-treated MSCs (*n* = 12). **c** In vitro levels of VEGF protein in MSC cultures in CM, PLF, and aCSF. Data presented with mean ± standard deviation. The Kruskal-Wallis test with Dunn’s test to correct for multiple comparisons were performed to determine statistical significance (* indicates *p* < 0.05; ** indicates *p* < 0.01; *** indicates *p* < 0.001; **** indicates *p* < 0.0001)

In examining the expression of immune-related cytokines, we found elevated expression of several factors in aCSF-treated MSCs as compared to PLF media-treated MSCs, including *IL6* and *CCL2* (encodes MCP-1) to levels comparable to CM-treated cells. MSCs lacked expression of pro-inflammatory factors. No expression was seen of *TNFα*, *IFNγ*, or *IL33* in any MSC culture condition, and *IL32* was reduced in aCSF (Fig. 4a; non-expressed factors not shown).

When we looked at the 281 and 392 genes that are significantly upregulated by aCSF-treated MSCs compared with PLF media-treated and CM-treated MSCs, respectively, we identified genes for several growth factors, including *BDNF*, *FGF1*, *FGF2*, *GDNF*, *NGF*, *PGF*, *VEGFA*, and *VEGFB* (Fig. 4b). Many of these factors promote angiogenesis, which was identified by functional annotation analysis as a top category of genes up-regulated in aCSF. Several of these factors are identified as angioneurins that have an effect on both angiogenesis and neuroprotection, such as VEGFA, NGF, and FGF [36].

ELISA assays were performed for VEGF on a separate set of MSC cultures exposed to CM, PLF, and aCSF. MSCs exposed to PLF had reduction in VEGF secretion when compared with CM. When exposed to aCSF, there was an initial drop in VEGF secretion at 24 h in culture, which recovered at 48 h (Fig. 4c).

### CSF growth factor protein levels are increased in humans after intrathecal MSC injection

**Fig. 5:**
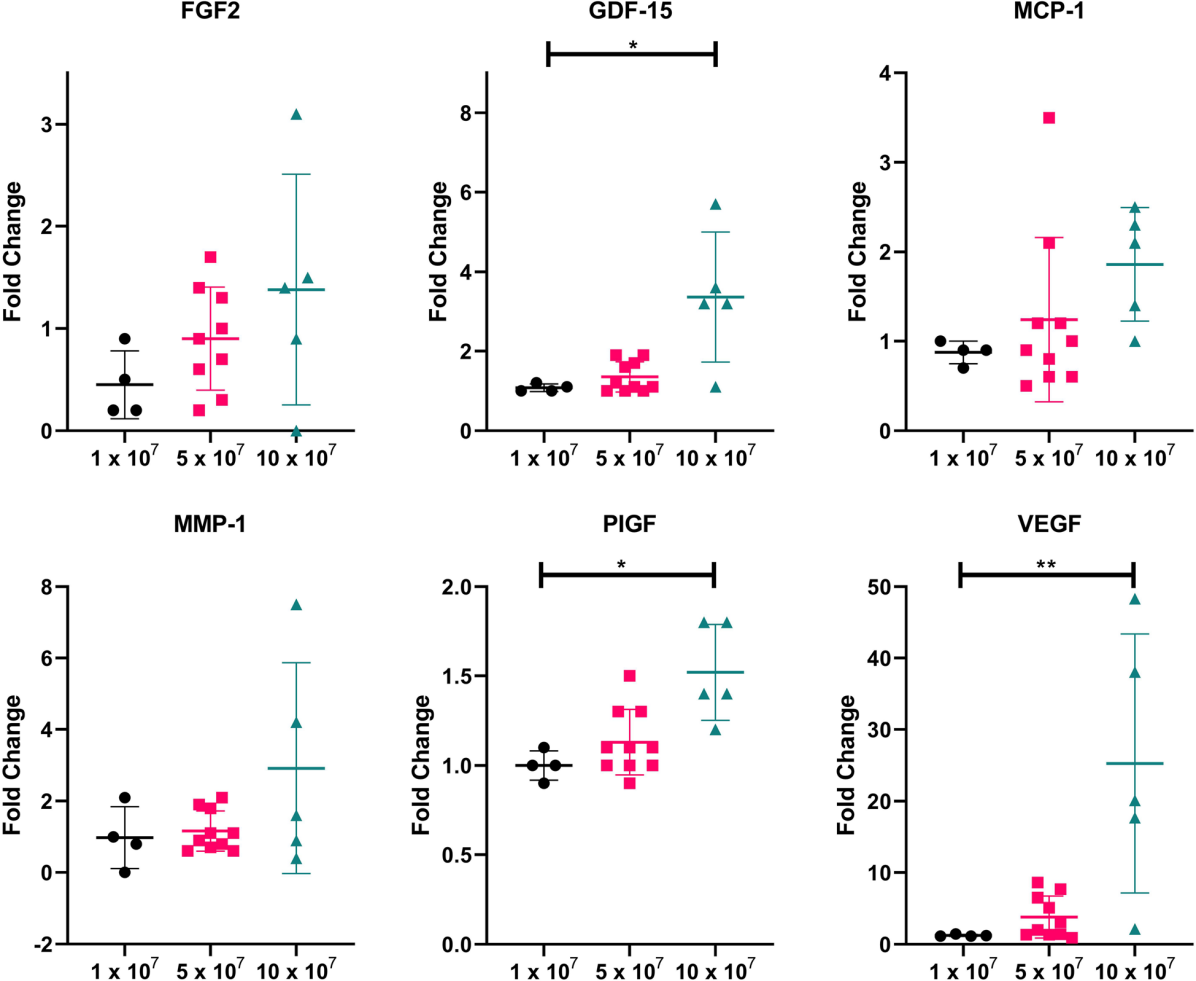
Fold change in protein level at 1 week post-intrathecal injections of MSCs. Patients with ALS received an intrathecal injection of either 1 × 10^7^, 5 × 10^7^, or 10 × 10^7^ autologous adipose-derived MSCs (*n* = 4, 10, and 5, respectively), and CSF samples were collected before MSC injection and at 1 week post-injection. The fold change of each factor was calculated for each patient, and the mean fold change ± standard deviations are plotted. The Kruskal-Wallis test with Dunn’s test to correct for multiple comparisons were performed to determine statistical significance (* indicates *p* < 0.05; ** indicates *p* < 0.01)

To further assess MSC behavior in CSF, protein quantitation was performed on CSF samples from subjects with ALS before and after intrathecal MSC administration. Subjects received an intrathecal dose of 1 × 10^7^, 5 × 10^7^, or 10 × 10^7^ autologous adipose-derived MSCs. The fold change in protein level comparing the 1 week time point to baseline levels was calculated for each patient and used for statistical analysis. A dose-dependent response trend was observed in VEGF protein levels for each group, with a 1.2 ± 0.14 fold change with 1 × 10^7^ MSCs, 3.8 ± 2.9 fold change with 5 × 10^7^ MSCs, and a significant 25.3 ± 18.1 fold change with 10 × 10^7^ MSCs (*p* = 0.006; Fig. 5). For PIGF and GDF-15, the 10 × 10^7^ MSC dose again correlates with a significant increase in protein levels compared to the 1 × 10^7^ and 5 × 10^7^ doses (*p* ≤ 0.03 for all; Fig. 5). Statistical analysis demonstrated that the 5 × 10^7^ dose did not correlate with significantly increased growth factor protein expression compared to the 1 × 10^7^ dose for VEGF, PIGF, or GDF-15. There was a trend for dose-dependent increases in MCP-1 and MMP-1, but this did not reach statistical significance. Altogether, high-dose intrathecal MSC injections resulted in increased levels of growth factors in the CNS compartment measured 1 week after treatment.

## Discussion

Our work expands the growing body of knowledge regarding the response characteristics of MSCs to novel stimuli or challenging environmental conditions, which, in turn, informs the use of MSCs in clinical applications [37]. While previous human in vivo studies have provided early evidence of therapeutic benefits in ALS following intrathecal MSC treatment [17, 38, 39], the long-term viability and mechanism of action of MSCs in the CSF environment remain unclear [40–42]. To determine how MSCs fare in the intrathecal space, we conducted a comprehensive investigation of the effects of CSF on human adipose-derived MSCs (manufactured with GMP-grade reagents) that examined changes in morphology, metabolism, and gene expression. Despite no clear gross morphological changes in MSCs cultured in CSF, there was a significant reduction in metabolic activity and a dramatically altered gene expression profile. Transcriptomic analyses revealed that MSCs increase expression of genes for growth factors and immune-modulating factors following culture in CSF. VEGF secretion was likewise maintained in aCSF. Remarkably, the transcriptomic analysis mirrored the protein changes in CSF from subjects with ALS that were treated with intrathecal MSCs.

The unbiased transcriptomic analysis also revealed that MSCs demonstrate a distinct, robust profile of gene expression when cultured in aCSF, PLF media, or CM. MSCs in aCSF and PLF retain the expression of genes encoding cell surface markers and reprogramming factors characteristic of MSCs in CM [20, 43], but lack expression of markers of active cellular proliferation. However, mRNA levels for markers of post-proliferative cells were differentially expressed to varying degrees in aCSF-treated MSCs as compared to PLF media-treated and CM-treated cells. The differential expression of Wnt ligands and pathway modulators suggests that aCSF-treated cells are quiescent, yet retain the ability to differentiate and promote synaptic plasticity, in part due to the expression of factors along the non-canonical Wnt pathway and the conserved expression of reprogramming factors [34, 35]. This expression pattern likely reflects that aCSF-treated MSCs—which decrease proliferation while sustaining trophic factor expression—are in a post-proliferative state that is distinct from that of hPL-deprived MSCs cultured in PLF media or confluent cells in CM.

When we examined genes that were significantly upregulated in aCSF compared to PLF media and CM, numerous genes for immune-modulating factors that are expressed at more than twice the levels seen in PLF media were identified. The expression of anti-inflammatory factors and lack of expression of pro-inflammatory genes by MSCs in aCSF suggests that these cells would suppress, rather than promote, inflammation in the CNS, consistent with previous reports suggesting this anti-inflammatory effect is therapeutic in disease models [41, 44]. Immune system abnormalities [45–47] and neuroinflammation [48, 49] are increasingly recognized as contributors to disease pathology in ALS and are being explored as therapeutic targets in clinical trials.

Strikingly, aCSF exposure augmented the expression of angiogenic and growth factor genes over and above expression levels seen in PLF media. Previous studies have reported that human MSCs, even under serum deprivation conditions, are highly angiogenic [50–53], and express growth factors that promote neuroprotection, remyelination, and therapeutic effects in animal models of neurological diseases [54–57]. In support of these prior studies, we found that cells incubated in aCSF express genes for angiogenic and growth factors (e.g., *GDNF*, *MCP-1*) at levels seen in CM-treated MSCs. Increased *MCP*-1 and *GDNF* expression by MSCs delivered intrathecally or intramuscularly into a rat model of ALS has been shown to delay motor function loss and to increase survival [41, 56]. In addition, this study found that aCSF caused expression of several genes for growth factors above the expression levels seen in CM (*BDNF*, *FGF2*, *NGF*, *PGF*, *VEGFA*, *VEGFB*).

This study identified that MSCs upregulate VEGF expression in both in vitro and in vivo conditions, giving insight to the probable mechanism of action of MSCs. Notably, upregulation of VEGF protein can regulate both angiogenesis and neurodegeneration, including degeneration of motoneurons [36]. For instance, studies have found that mice had reduced VEGF levels prior to developing symptoms of spinal and bulbar muscular atrophy and adult-onset motoneuron degeneration that resembled the degeneration seen in ALS [58, 59]. Conversely, overexpression of VEGF improves motor performance and prolongs survival in rodent models of ALS, particularly when paired with enhanced GDNF expression [60]. Likewise, expression of VEGF promotes neurogenesis and neural stem cell differentiation post-injury [61, 62]. By increasing neural perfusion around sites of neurodegeneration and ischemia, VEGF could promote neuroprotection for surviving motoneurons in the spinal cord and stimulate neuroregeneration. This hypothesis is further supported by the early success of clinical trials using MSCs with enhanced VEGF secretion to treat neurodegenerative disorders [11, 38]. We measured a dose-dependent increase in CSF VEGF in patients with ALS following intrathecal treatment of autologous adipose-derived MSCs. The combination of increased gene expression in in vitro conditions and increased protein levels in in vivo conditions support the claim that MSCs within the intrathecal space produce significant quantities of therapeutic growth factors. Alternatively, MSCs could induce CNS cells to upregulate growth factor production. Regardless of the source of growth factors, their increased quantities confirm that MSCs are exerting therapeutic effects within the CNS compartment. Furthermore, high doses of MSCs (10 × 10^7^) were required to elicit significant increases in protein expression of beneficial growth factors. These data are critical for researchers using MSC-based treatments as there are very few studies comparing MSC doses on therapeutic effect [16, 17].

There are now several completed human clinical trials that have utilized intrathecal autologous MSC therapy for ALS [17, 38, 39, 63]. While there are signs of efficacy of MSC treatment in ALS, it is also becoming clear that some patients are more or less likely to respond to this therapy. Understanding this variability in response to MSC therapy will be critical for understanding ALS pathogenesis, MSC therapies, and how best to translate this therapy to clinical practice. Berry et al. reported that higher CSF VEGF was correlated with lower CSF MCP-1 in patients treated with modified bone-marrow-derived MSCs [38]. They also reported that lower post-MSC treatment MCP-1 correlated with better outcomes, which was also seen in Oh et al. [39]. Our phase I study did not have clinical outcomes to correlate with the CSF findings; however, we are now pursuing these hypotheses as part of an active phase II clinical trial of MSCs in ALS (ClinicalTrials #NCT03268603).

To our knowledge, this is the first reported large-scale transcriptomic analysis of multiple lines of human adipose-derived MSCs exposed to undiluted aCSF or hCSF. Unbiased examination of the differential expression of over 23,000 genes provided the most detailed understanding to date of how the transcriptomic profile of clinical-grade MSCs varies between normal, hPL-deprived, and intrathecal-like culture conditions. By understanding how the response of MSCs to CSF resembles—and is distinct from—the response to CM or PLF media, the field gains an appreciation for the scope of the phenotypic responses of MSCs. We further have shown that intrathecal injection of adipose-derived autologous MSCs in subjects with ALS leads to a dose-dependent increase in CSF growth factors. From a therapeutic standpoint, if MSCs respond to individual culture conditions with unique gene expression profiles, then proper preparation of MSCs could impact the secretion of therapeutic factors and biomarkers pertinent to the response of patients to MSC treatment.

## Conclusion

Our results demonstrate that MSCs can remain viable and morphologically normal in CSF. Under intrathecal conditions, the cells will decrease metabolic activity and will alter their gene expression to adapt to the novel environment while maintaining high-level expression of supportive factors that can be measured in the CSF of subjects treated with intrathecal MSCs for ALS. Overall, our results identify potential modes of therapeutic efficacy as well as candidate biomarkers of MSC potency to guide clinical translation of MSC-based therapies for neurodegenerative diseases.

This work validates the continued use MSCs in clinical trials, and also supports using the 10 × 10^7^ dose to maximize therapeutic benefit.

## Supplementary Information

**Additional file 1: Supplemental Figure 1**. Expression levels of detectable transcripts of lineage-specific genes for adipogenesis, chondrogenesis, and osteogenesis. Expression levels of transcripts encoding markers of MSC lineage differentiation (*n* = 12, data shown with median and interquartile range).

**Additional file 2: Supplemental Figure 2**. Expression levels of detectable transcripts of genes associated with the canonical and non-canonical Wnt signaling pathways. Expression levels of transcripts encoding markers of Wnt pathway signaling (n = 12, data shown with median and interquartile range).

**Additional file 3: Supplemental Figure 3**. Expression levels of selected genes using real-time qPCR confirms transcriptonic dataset.

## Data Availability

We will be submitting our transcriptomics data to Gene Expression Omnibus (GEO) in line with the NIH Genomic Data Sharing (GDS) Policy.

## Abbreviations

MSC: Mesenchymal stromal cell
CSF: Cerebrospinal fluid
hCSF: Human cerebrospinal fluid
aCSF: Artificial cerebrospinal fluid
ALS: Amyotrophic lateral sclerosis
CNS: Central nervous system
hPL: Human platelet lysate
GMP: Good manufacturing practices
CM: Control media
PLF: Platelet lysate-free
IRB: Institutional Review Board
MEM: Minimal essential medium
PBS: Phosphate buffer solution
PCR: Polymerase chain reaction
FPKM: Fragments per kilobase pair per million mapped reads
