# Supplementary figures and images for: Alterations of mesenchymal stromal cells in cerebrospinal fluid: insights from transcriptomics and an ALS clinical trial
Source: Stem Cell Res Ther. 2021 Mar 18;12:187. doi: 10.1186/s13287-021-02241-9 (PMC7977179; doi:10.1186/s13287-021-02241-9)

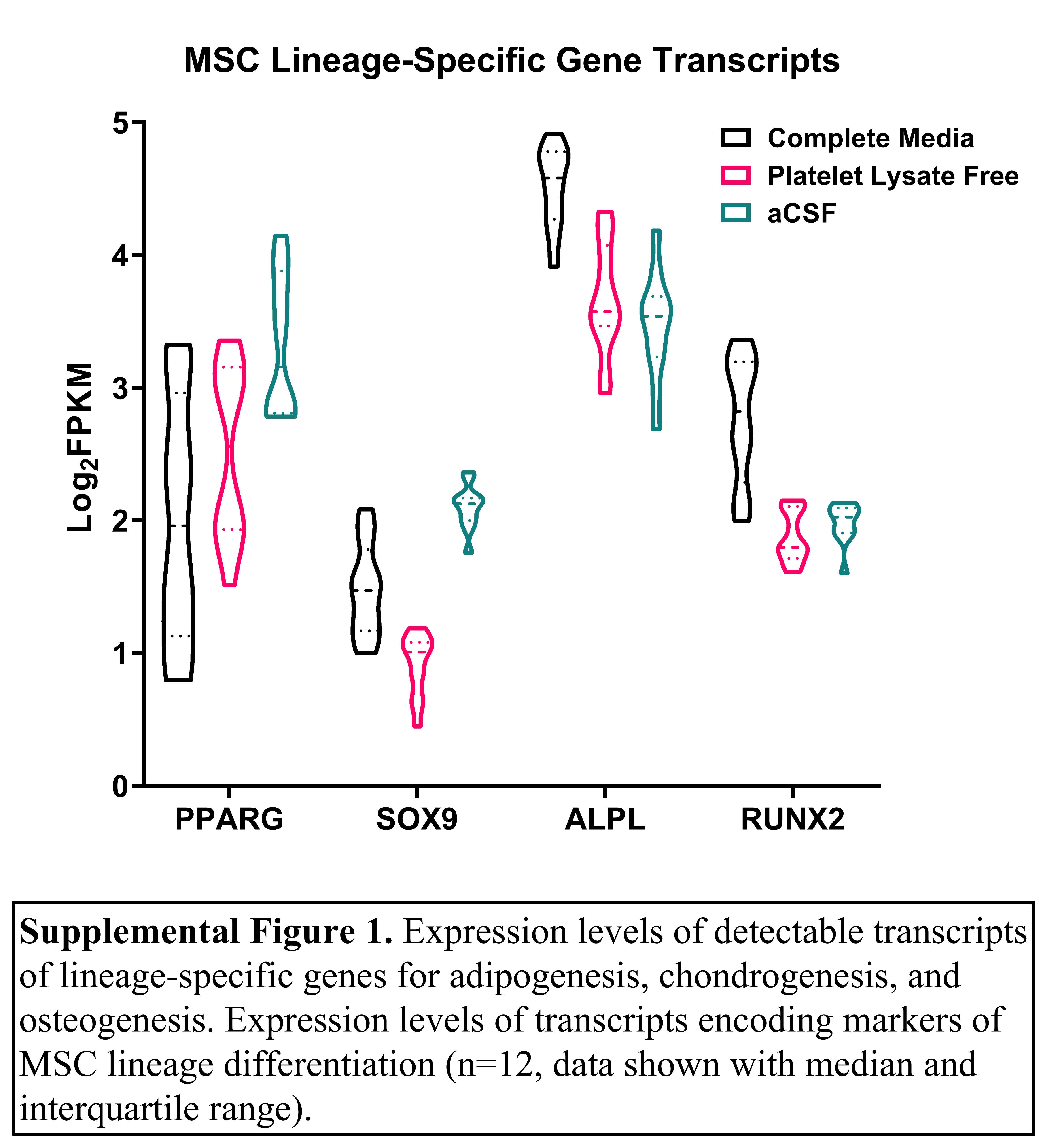

Supplement: Supplementary file 1 — Additional file 1: Supplemental Figure 1. Expression levels of detectable transcripts of lineage-specific genes for adipogenesis, chondrogenesis, and osteogenesis. Expression levels of transcripts encoding markers of MSC lineage differentiation (n = 12, data shown with median and interquartile range). [file 13287_2021_2241_MOESM1_ESM.jpg]

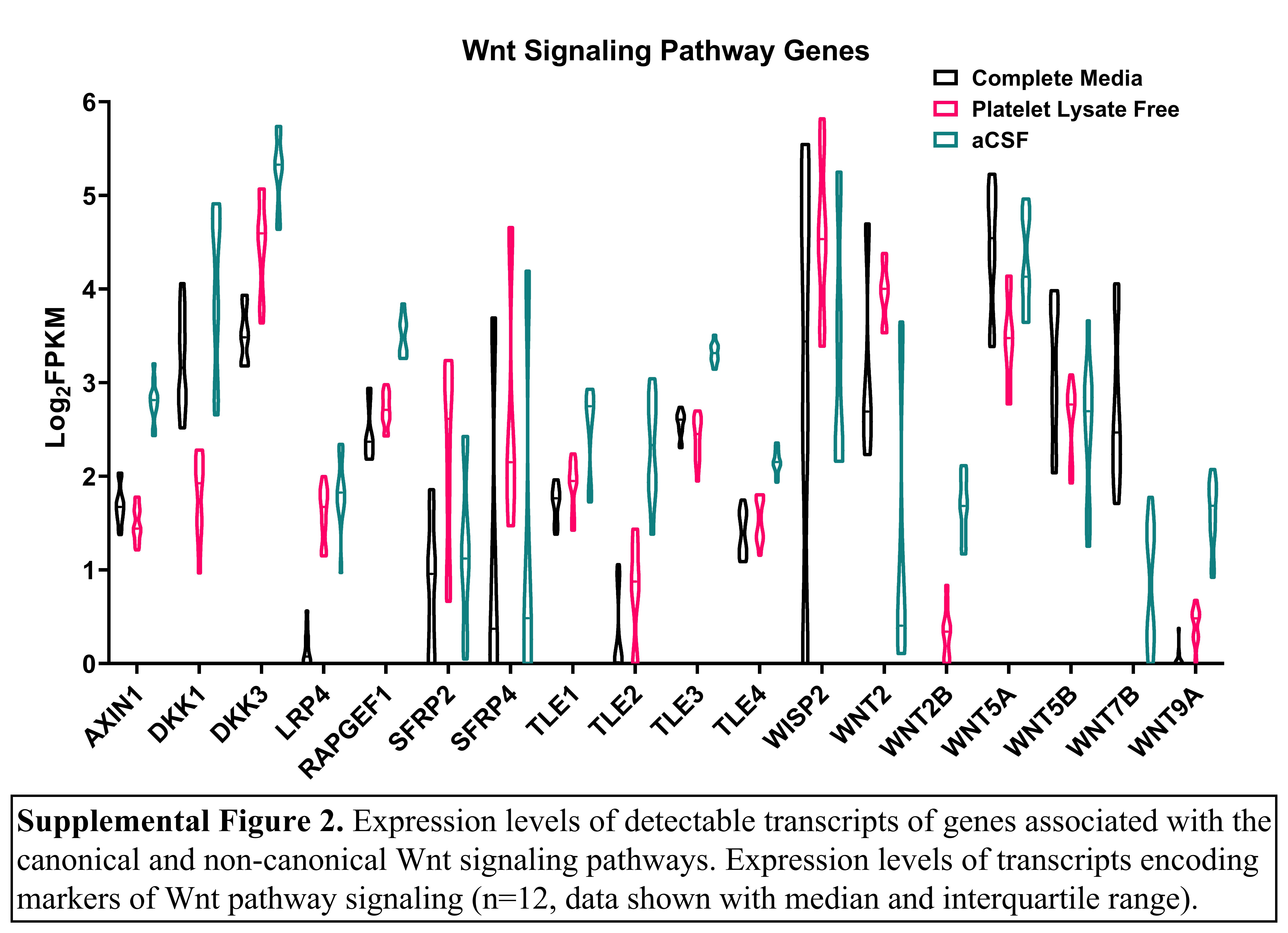

Supplement: Supplementary file 2 — Additional file 2: Supplemental Figure 2. Expression levels of detectable transcripts of genes associated with the canonical and non-canonical Wnt signaling pathways. Expression levels of transcripts encoding markers of Wnt pathway signaling (n = 12, data shown with median and interquartile range). [file 13287_2021_2241_MOESM2_ESM.jpg]

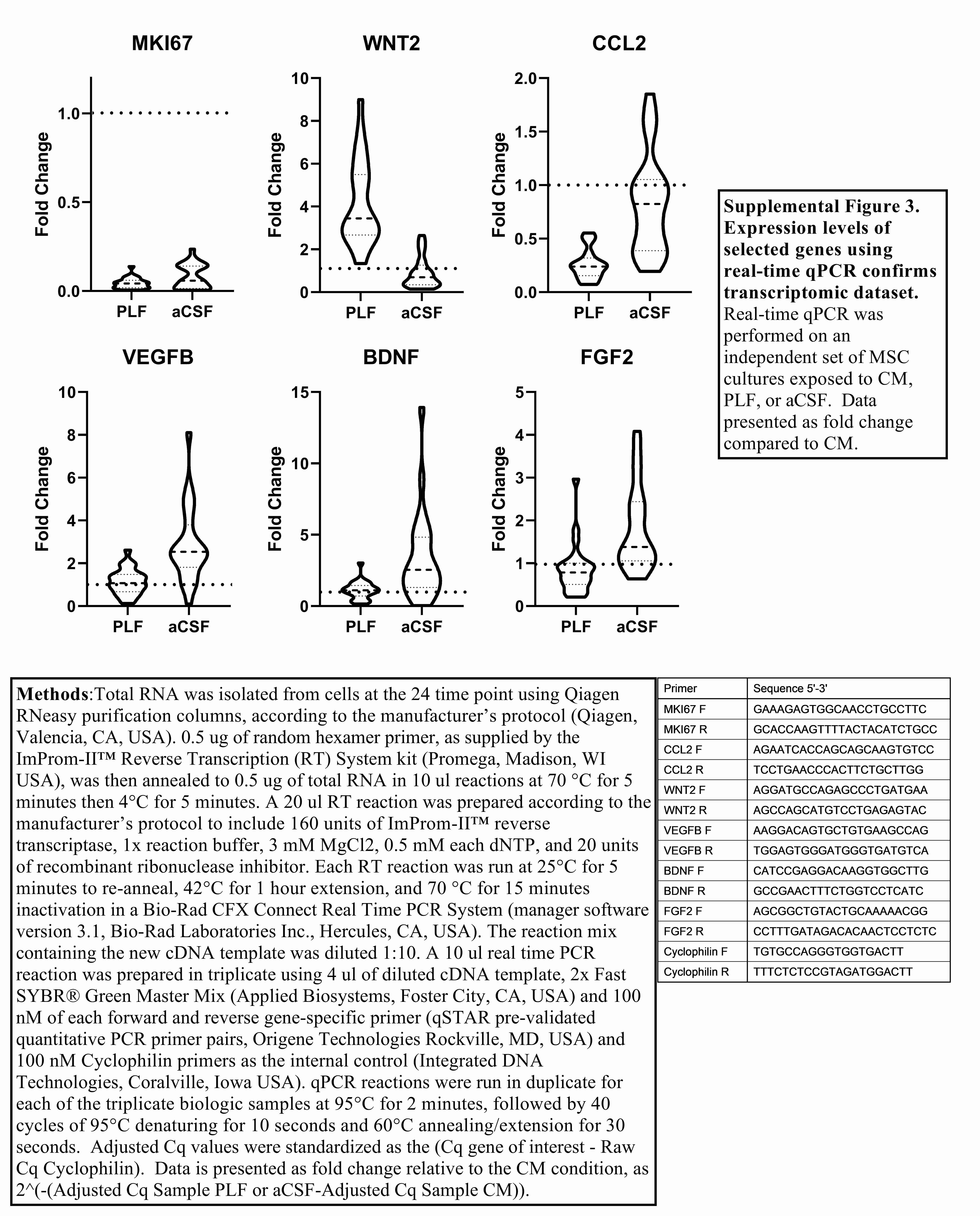

Supplement: Supplementary file 3 — Additional file 3: Supplemental Figure 3. Expression levels of selected genes using real-time qPCR confirms transcriptonic dataset. [file 13287_2021_2241_MOESM3_ESM.jpg]
